# Association of health literacy and general self-efficacy with emergency department visits for unclear abdominal pain after bariatric surgery

**DOI:** 10.1007/s00423-025-03736-2

**Published:** 2025-05-17

**Authors:** Jenny Angerås-Kraftling, Maria Jaensson, Karuna Dahlberg, Erik Stenberg

**Affiliations:** 1 Department of Surgery, Faculty of Medicine and Health, Örebro University, Örebro, Sweden; 2 Faculty of Medicine and Health, School of Health Sciences, Örebro University, Örebro, Sweden

**Keywords:** Obesity, Bariatric surgery, Health literacy, General self efficacy, Adverse outcome, Emergency room visits

## Abstract

**Introduction:**

Emergency department visits are common following bariatric surgery and may be partially preventable. Health literacy and general self-efficacy are factors that may influence health-seeking behaviors in these patients. This study aimed to assess whether health literacy and general self-efficacy are associated with an increased frequency of emergency department visits after bariatric surgery.

**Methods:**

Patients who underwent bariatric surgery at a single hospital from 2018 to 2020 were evaluated for their health literacy and general self-efficacy levels before surgery. Data on emergency department visits within the patient’s residential region were evaluated over a three-year period, with repeated emergency department visits for abdominal pain as the primary outcome.

**Results:**

During the follow-up period, 69 of 231 patients (29.9%) had at least one emergency department visit for abdominal pain, and 20 patients (8.7%) had three or more visits. Inadequate functional health literacy (OR 5.56, 95% CI 1.80-17.19, *p* = 0.003) and inadequate communicative and critical health literacy (OR 10.48, 95% CI 3.13–35.08, *p* < 0.001) were both significantly associated with an increased risk of repeated emergency department visits over the three-year period. No significant association was found between low general self-efficacy and the frequency of emergency department visits.

**Conclusions:**

Inadequate health literacy is associated with an increased risk of repeated emergency department visits for abdominal pain following bariatric surgery.

**Supplementary Information:**

The online version contains supplementary material available at 10.1007/s00423-025-03736-2.

## Introduction

Obesity prevalence has surged in recent decades, making it a major global public health threat [1]. While pharmacological treatments show promising results, metabolic and bariatric surgery (MBS) remains the most effective treatment, leading to significant weight loss and improvements in metabolic comorbidities and health-related quality of life for most patients [2–4]. However, emergency department visits are common, with nausea and abdominal pain being the most frequent causes. Although serious postoperative complications are possible, many of these return visits to the emergency department are potentially preventable [5, 6].

Health literacy has been shown to influence postoperative recovery [7–9]. Low health literacy is associated with poor compliance with recommendations following emergency general surgery, increasing the risk of readmission, complications, and prolonged functional recovery [10]. While limited health literacy might contribute to problematic recovery, including emergency department visits, a prior smaller study did not find support for such an association [11]. Self-efficacy, defined as an individual’s belief in their ability to achieve a desired goal or outcome, interacts with self-care management [12]. Therefore, this study aims to explore whether health literacy and general self-efficacy are associated with increased emergency department utility after MBS.

## Methods

The study is a subgroup analysis of a prospective, longitudinal, mixed-method study conducted at three hospitals in Sweden. Adults (≥ 18 years of age) scheduled for primary Roux-en-Y gastric bypass or sleeve gastrectomy, with the ability to read and understand Swedish, were considered for inclusion [13].

Prior to surgery, patients completed three translated and validated questionnaires assessing functional health literacy (FHL), communicative and critical health literacy (C&CHL), and general self-efficacy. The current study included patients who underwent surgery at one hospital (Lindesberg Hospital). A surgeon (J.A.), not involved in patient care, reviewed the charts for all emergency department visits within the region, Region Örebro County. The reasons for seeking emergency care, diagnoses, and results of further examinations were evaluated, with unclear causes of abdominal pain considered when no clear cause was identified. Any uncertainties were further evaluated by another surgeon (ES) blinded to the patients’ baseline characteristics.

### Definitions

Baseline characteristics were recorded before initiating the preoperative weight-reducing diet. Comorbidities were defined as obesity-related conditions (type-2 diabetes, hypertension, dyslipidemia, sleep apnea and depression) requiring pharmacological treatment, nocturnal continuous positive airway pressure (CPAP), or nocturnal bilevel positive airway pressure (BiPAP). Postoperative complications were defined and classified according to the Clavien-Dindo classification of postoperative complication as those occurring within 30 days of surgery, with serious complications being those requiring intervention under general anesthesia or resulting in organ failure or death [14].

### The functional health literacy scale

FHL focus on understanding health-related information [15]. The Swedish version of this scale has documented high reliability and validity for a Swedish population undergoing MBS [16, 17]. It consists of five items rated on a 5-point Likert scale, with higher scores indicating sufficient health literacy and lower scores indicating problematic or inadequate health literacy [16].

### The communicative and critical health literacy scale

This scale assesses more complex skills, including the ability to extract, analyze, and communicate health information [15].The scale has also been reported to have high reliability and validity in the Swedish MBS population [17, 18]. It consists of five items rated on a 5-point Likert scale, with higher scores indicating sufficient health literacy, and lower scores indicating problematic or inadequate health literacy [18, 19].

### The general Self-Efficacy scale

This scale measures the belief in one’s ability to accomplish specific goals. It has been validated in the Swedish MBS population and reported to be reliable and valid [20–22]. It consists of 10 items rated on a 4-point Likert scale, with lower scores indicating lower self-efficacy [20]. A predefined cutoff score of 30 was used to categorize high and low general self-efficacy [23].

### Surgery

All patients underwent either laparoscopic sleeve gastrectomy or Roux-en-Y gastric bypass procedures, with perioperative care in accordance with the Enhanced Recovery after Surgery (ERAS) protocol [24]. For Roux-en-Y gastric bypass, an antecolic, antegastric technique was used, with a 100–120 cm alimentary limb and a 50–80 cm biliopancreatic limb. A short gastric pouch was constructed via a linear stapling technique with a hand-sewn remaining defect. Mesenteric defects were routinely closed using either running, non-absorbable sutures or non-resorbable clips at the discretion of the surgeon. Sleeve gastrectomy involved resection starting < 5 cm from the pylorus, ending 1 cm from the angle of His using a 35Fr Bougie in all cases. Stapler line reinforcement was not routinely used, but the stapler line was reinforced partly or completely via resorbable sutures at the discretion of the surgeon. The protocol included routine discharge at postoperative Day 1 if oral intake was adequate and no complications were detected. Routine follow-up occurred at Day 30, 6 months, and 1 and 2 years after surgery.

### Outcomes

The main outcome for the present study was repeated emergency department (3 or more) visits during the first 3 years after surgery. The secondary outcome was any visit to the emergency department with or without a diagnosis.

### Statistics

Continuous data exhibited a normal distribution and were presented as the means ± standard deviations (SDs). Categorical data were presented as numbers and proportions. The risk for repeated emergency department visits was evaluated via logistic regression adjusted for surgical method. SPSS version 29 (IBM, Armonk, NY, USA) was used for the statistical analyses. A two-sided p-value < 0.05 was considered to represent statistical significance.

### Ethics

The study was approved by the Regional Ethics Board in Uppsala, Sweden (Ref: 2018/256) and conducted in accordance with the standards of the 1964 Helsinki declaration and its later amendments.

## Results

From October 15, 2018 until November 3, 2020, 686 patients were included in the study. Among these patients, 240 underwent surgery at Lindesberg Hospital. After the exclusion of two patients who died and 7 patients who moved to another county during the follow-up period, 231 patients (96%) remained in this study. In the study group, 135 patients underwent Roux-en-Y gastric bypass (58%) and 96 underwent sleeve gastrectomy (42%). The baseline characteristics of the patients in the study group are presented in Table 1. Postoperative complications within the first 30 days occurred in 12 patients (5.2%), 6 of whom were considered serious complications (2.6%). The absolute number of complications was too small to be stratified on the basis of health literacy or general self-efficacy.

**Table 1:** Baseline characteristics

	Missing data, *n* (%)	*n* (%)
Numbers, n		231
Age	0	41.4 ± 11.14
Baseline BMI	0	41.8 ± 5.73
Waist circumference	1 (0.4%)	123.1 ± 13.57
Sex	0	
Women		168 (72.7%)
Men		63 (27.3%)
Comorbidities	0	
Type-2 diabetes		27 (11.7%)
Hypertension		66 (28.6%)
Dyslipidemia		10 (4.3%)
Sleep apnea		32 (13.9%)
Depression		22 (9.5%)
Smoking	8 (3.5%)	
Active smoking		23 (10.3%)
History of smoking		91 (40.8%)
Never smoked		109 (48.9%)
Education	1 (0.4%)	
Primary school		16 (7.0%)
Secondary school		169 (73.2%)
Higher education		45 (19.5%)
FHL	2 (0.9%)	
Inadequate		36 (15.6%)
Problematic		86 (37.2%)
Sufficient		107 (46.3%)
C & C HL	11 (4.8%)	
Inadequate		15 (6.5%)
Problematic		74 (32.0%)
Sufficient		131 (56.7%)
GSE	12 (5.2%)	
Low		82 (37.4%)
High		137 (62.6%)

FHL = Functional health literacy, C & C HL = Communicative and critical health literacy, GSE = General self-efficacy, n = number

### Emergency department visits

During the three-year follow-up, 69 patients (29.9%) had a total of 175 emergency department visits, with the most common diagnosis being gallstone disease (*n* = 28, 16%), followed by dumping/hypoglycemia (*n* = 16, 9%), infection (*n* = 8, 5%), abdominal wall-related pain (*n* = 7, 4%), bowel obstruction (*n* = 6, 3%), ureterolithiasis (*n* = 6, 3%), marginal ulcers (*n* = 6, 3%) and appendicitis (*n* = 6, 3%; Supplementary Table 1). No diagnosis was found at 75 visits (43%). In total, 37 patients had at least one visit for unclear abdominal pain (16.0%). Three or more visits were reported for 20 patients (8.7%).

A greater proportion of patients with inadequate FHL and C&CHL visit the emergency department for abdominal pain (Table 2) and more often visit without reaching a diagnosis (Table 3).

**Table 2:** Any emergency department visits stratified according to preoperative health literacy and general self efficacy

	*N* (%)	OR (95%CI)*	P*
FHL			
Inadequate	18 (50.0%)	2.57 (1.17–5.63)	0.019
Problematic	20 (23.3%)	0.73 (0.38–1.40)	0.345
Sufficient	31 (29.0%)	Ref	Ref
C & C HL			
Inadequate	10 (66.7%)	4.24 (1.36–13.24)	0.013
Problematic	14 (18.9%)	0.47 (0.23–0.93)	0.030
Sufficient	43 (32.8%)	Ref	Ref
GSE			
Low	28 (34.1%)	1.36 (0.75–2.45)	0.313
High	38 (27.7%)	Ref	Ref

FHL = functional health literacy; C&C HL = communicative and critical health literacy; GSE = general self-efficacy; OR = odds ratio; 95% CI = 95% confidence interval

*adjusted for surgical method

**Table 3:** Emergency department visits without diagnosis stratified on preoperative health literacy and general self efficacy

	*N* (%)	OR (95%CI)*	P*
FHL			
Inadequate	15 (41.7%)	4.68 (1.95–11.25)	< 0.001
Problematic	8 (9.3%)	0.69 (0.27–1.72)	0.421
Sufficient	14 (13.1%)	Ref	Ref
C & C HL			
Inadequate	7 (46.7%)	5.05 (1.63–15.60)	0.005
Problematic	10 (13.5%)	0.94 (0.41–2.14)	0.937
Sufficient	19 (14.5%)	Ref	Ref
GSE			
Low	13 (15.9%)	0.92 (0.44–1.94)	0.827
High	23 (16.8%)	Ref	Ref

FHL = functional health literacy; C&C HL = communicative and critical health literacy; GSE = general self-efficacy; OR = odds ratio; 95% CI = 95% confidence interval

*adjusted for surgical method

Inadequate FHL and CCHL were associated with greater proportion of patients with repeated emergency departments visits, while no difference was observed according to the general self-efficacy (Table 4).

**Table 4:** Repeated emergency department visits (3 or more) stratified according to preoperative health literacy and general self efficacy

	*N* (%)	OR (95%CI)*	P*
FHL			
Inadequate	9 (25.0%)	5.56 (1.80-17.19)	0.003
Problematic	5 (5.8%)	1.04 (0.31–3.55)	0.946
Sufficient	6 (5.6%)	Ref	Ref
C & C HL			
Inadequate	7 (46.7%)	10.48 (3.13–35.08)	< 0.001
Problematic	3 (4.1%)	0.52 (0.14–1.94)	0.327
Sufficient	10 (7.6%)	Ref	Ref
GSE			
Low	6 (7.3%)	0.82 (0.29–2.27)	0.696
High	12 (8.8%)	Ref	Ref

FHL = functional health literacy; C&C HL = communicative and critical health literacy; GSE = general self-efficacy; OR = odds ratio; 95% CI = 95% confidence interval

*adjusted for surgical method

## Discussion

Inadequate functional and communicative and critical health literacy, but not general self-efficacy, were associated with increased emergency department visits during the first three years post-surgery.

Emergency department visits for abdominal pain were frequent, with 30% of patients seeking care for this issue on one or more occasions. Most previous studies have focused on emergency department visits 30–90 days post-surgery, but two prior studies reported results consistent with the present study [25, 26]. Despite receiving pre- and postoperative information, many patients sought emergency care due to a perceived sense of urgency in symptoms [27]. However, many of these visits did not result in readmission or advanced intervention, suggesting these visits were avoidable [26, 28].

Post-surgery, many patients struggle to regain a sense of normality; and to retain control of their situation [29]. Some are also struggling with receiving adequate support, particularly for psychosocial challenges [30]. Those with inadequate health literacy may experience difficulties in navigating recovery, including understanding and meeting the challenges and demands after a weight-loss intervention [31]. This may explain prolonged recovery after day surgery [32], as well as increased risk for weight regain after MBS reported among patients with reduced health literacy [33]. Limited C&C HL impedes effective communication with health care providers, finding and evaluating reliable information, and navigating the health care system [18, 19]. For some, the emergency department becomes the easiest way to seek care.

In contrast, general self-efficacy, which pertains to one’s belief in their ability to organize and execute actions to achieve certain goals, did not appear to influence emergency department visits in this study. While there may be mediating effects between health literacy and general self-efficacy, the two constructs are not directly related. Based on the results of this study, the general self-efficacy may be less influential in health-seeking behavior after MBS.

Patients with reduced health literacy face challenges in accessing, understanding and applying health-related information [34]. Despite comprehensive pre- and postoperative information and support protocols, information and follow-up may need to be further tailored to meet the capacity and need of these patients. Prior studies suggest that these individuals require individualized information, especially given the difficulties with reading and understanding written texts they may face [16, 35]. Although telemedicine interventions have been received well and may reduce the risk for attrition, they have not been shown to reduce emergency department visits within 30 days post-surgery [36]. Further research is needed to identify the specific requirements and needs of patients with inadequate health literacy, to guide interventions aimed at improving MBS outcomes for this group.

The study’s strengths include the use of validated scales for health literacy and general self-efficacy, as well as the very high follow-up rate over three years after surgery. However, with an observational study design, the study cannot address direct causation, and any conclusion will be limited to associations. Furthermore, the study was conducted in one region in Sweden and included only patients who could read and write in the Swedish language. The study group is similar to that seen in most other northern European countries, but the results may not be representative of other communities.

## Conclusion

Inadequate health literacy is associated with an increased risk for repeated emergency department visits for abdominal pain following bariatric surgery.

## Electronic supplementary material

Below is the link to the electronic supplementary material.

Supplementary Material 1

## Data Availability

Data cannot be shared publicly because of patient confidentiality under current Swedish legislation. Data are available from the Scandinavian Obesity Surgery Registry (contact via soreg@regionorebrolan.se), the National Diabetes Register (ndrinfo@registercentrum.se) and the Swedish Board of Health and Welfare (contact via Registerservice@socialstyrelsen.se) upon reasonable request.

## Declarations

### Preregistration plan

No preregistration exists for the study reported in this article.

### Twitter summary

Reduced health literacy associated with higher risk for emergency department visits for abdominal pain up to 3 years after bariatric surgery @ErikStenberg_MD.
