# Supplementary material for: Association of health literacy and general self-efficacy with emergency department visits for unclear abdominal pain after bariatric surgery
Source: Langenbecks Arch Surg. 2025 May 17;410(1):162. doi: 10.1007/s00423-025-03736-2 (PMC12085352; doi:10.1007/s00423-025-03736-2)
Supplement: Supplementary file 1 — Supplementary Material 1 [file 423_2025_3736_MOESM1_ESM.docx]

| **Supplementary Table 1**. Diagnosis at emergency department visit | |
| --- | --- |
| Diagnosis | N (%) |
| Gallstone disease | 28 (16%) |
| Dumping/hypoglycemia | 16 (9%) |
| Infection | 8 (5%) |
| Abdominal wall related pain | 7 (4%) |
| Bowel obstruction | 6 (3%) |
| Ureterolithiasis | 6 (3%) |
| Appendicitis | 6 (3%) |
| Gastric ulcer | 6 (3%) |
| Sleeve stenosis | 5 (3%) |
| Gastrointestinal bleeding | 4 (2%) |
| Gastroenteritis | 3 (2%) |
| Pregnancy-related pain | 2 (1%) |
| Other gynecological reason | 1 (1%) |
| Diverticulitis | 1 (1%) |
| Unclear diagnosis | 1 (1%) |
| No diagnosis | 75 (43%) |
